# Effects of Different Components of Buyang Huanwu Tang on the PPAR*γ*/LXR*α*/ABCA1 Pathway in Hypercholesterolemia Mouse Model

**DOI:** 10.1155/ijog/9595757

**Published:** 2025-07-09

**Authors:** Shuaihu Yang, Yukun Zhang, Xinxin Liu, Xingtong Chen, Yuxue Ma, Shijian Fang, Ruihong Yang, Jinbiao Yang, Yunyue Zhou, Xiao He, Pengcheng Li, Hongbin Xiao, Wenying Niu

**Affiliations:** ^1^College of Basic Medicine, Heilongjiang University of Traditional Chinese Medicine, Harbin, Heilongjiang, China; ^2^College of Traditional Chinese Medicine, Ningxia Medical University, Yinchuan, Ningxia, China

**Keywords:** 30% ethanol elution part, 50% ethanol elution part, 75% ethanol elution part, ABCA1, Buyang Huanwu Tang, cholesterol reverse transport, ethanol-deposited part, hypercholesterolemia, LXR*α*, PPAR*γ*

## Abstract

The aim of this study is to compare the effects of different components of Buyang Huanwu Tang (BYHWT) on the peroxisome proliferator-activated receptor *γ* (PPAR*γ*)/liver X receptor *α* (LXR*α*)/ATP-binding cassette transporter A1 (ABCA1) pathway and its lipid-lowering effects. This study shows that the BYHWT alcohol precipitation and 75% alcohol components can significantly reduce the serum levels of triglycerides (TGs), low-density lipoprotein (LDL), cholesterol (CHO), and hepatic function damage indicators such as glutamic oxaloacetic transaminase (AST) and glutamic pyruvic transaminase (ALT) in hypercholesterolemia mouse model. After treatment, the presence of lipid droplets in liver cells was reduced, and the destruction of adipocytes was improved. The Western blot (WB) results showed that alcohol precipitation and 75% alcohol components can upregulate PPAR*γ*, ABCA1, and LXR*α*. The expression of these components indicates that PPAR*γ* upregulation can activate LXR*α*, thus regulating the expression of ABCA1, mediating CHO efflux, promoting reverse cholesterol transport (RCT), and regulating the downstream gene CYP7A1 to participate in bile acid synthesis and metabolism. In summary, the experimental results indicate that the BYHWT alcohol precipitation, 50% alcohol, and 75% alcohol components can modulate the PPAR*γ*/LXR*α*/ABCA1 pathway in hypercholesterolemia mouse model to promote CHO metabolism.

## 1. Introduction

In recent years, with improvements in living standards and changes in diet structure, the prevalence of hypercholesterolemia has increased dramatically worldwide. It is estimated that the global prevalence of hypercholesterolemia in 2019 was approximately 15.2%. Moreover, the prevalence of hypercholesterolemia in adults in China has reached 40.4% while that in adults in some developed countries has reached 55%, making hypercholesterolemia a global public health event [1]. The pathogenesis of hypercholesterolemia is an extremely complex metabolic process. In hypercholesterolemia, triglycerides (TGs), low-density lipoprotein (LDL), cholesterol (CHO), and high-density lipoprotein levels are elevated. However, HDL levels are characteristically decreased [2]. Due to the accumulation of lipids, insulin resistance, and increase in oxidative stress in the body [3], fatty liver, coronary atherosclerosis, diabetes, and many other diseases can ultimately result [4]. At present, the chemical drugs commonly used in clinical practice to treat hypercholesterolemia can be roughly divided into three categories, namely, statins (atorvastatin), fibrates (fenofibrate), and CHO absorption inhibitors (ezetimibe). These drugs can effectively reduce the severity of hypercholesterolemia, but they need to be taken for a long time, and most are dependent and have substantial side effects. Statins often cause elevated levels of aminotransferase in patients with sensitivity, resulting in liver function damage, which can be gradually recovered after drug withdrawal. Moreover, long-term statin use can also cause autoimmune necrotizing myopathy [5]. Fibrate drugs cause loss of appetite, nausea and vomiting, and other gastrointestinal symptoms [6], while CHO absorption inhibitors cause cognitive dysfunction [7].

Buyang Huanwu Tang (BYHWT) comes from the “Medical Forest Correction” written by Wang Qingren in the Qing Dynasty. From the theory of Chinese medicine, BYHWT compatibility is characterized by a large quantity of Qi-supplementing drugs and a small amount of drugs with blood-activating, blood Qi-flourishing, blood activation without injury, and Qi-supplementing and blood-activating collateral properties. BYHWT can improve lipid metabolism during stable angina pectoris in elderly coronary heart disease patients with Qi deficiency and blood stasis [8]. In 60 patients with stable angina pectoris and coronary heart disease due to Qi deficiency and blood stasis, blood lipids were significantly reduced after the addition and reduction of BYHWT and Danshen Yin [9]. Fu et al. [10] proved that BYHWT can significantly upregulate the expression levels of the protein SREBP-2 and LDL in the liver tissue of hypercholesterolemia model golden hamsters; reduce the levels of TC, TG, and LDL; significantly increase the level of HDL; reduce the formation of fat vacuoles in liver tissue; and reduce lipid deposition. Fu et al. found that the glycosides in BYHWT could reduce TC, TG, and LDL in hypercholesterolemia mice, alleviate atherosclerosis symptoms, and inhibit the release of adhesion molecules and the activation of inflammatory pathways.

Modern pharmacology data show that the compounds *Astragalus*, kaempferol, and gallic acid [11–14] in BYHWT can regulate blood lipid levels. However, there are few studies on the effects of different components of BYHWT on the peroxisome proliferator-activated receptor *γ* (PPAR*γ*)/liver X receptor *α* (LXR*α*)/ATP-binding cassette transporter A1 (ABCA1) pathway. Therefore, this study was mainly conducted to confirm that different components of BYHWT reduced blood lipid levels in hypercholesterolemia mouse model through the PPAR*γ*/LXR*α*/ABCA1 pathway.

## 2. Materials

### 2.1. Animals

Eighty male ICR mice were provided by Liaoning Changsheng Biotechnology Co., Ltd. and the Experimental Animal Center of Heilongjiang University of Chinese Medicine with Animal Licence Number SCXK(Liao) 2020-0001. The animals were reared in a room at room temperature (20°C~24°C) with a relative humidity of 50%~60% on a 12 h circadian rhythm with free access to drinking water and solid feed. All animal studies were approved by the Experimental Animal Ethics Committee of Heilongjiang University of Chinese Medicine (Approval Number 2024050102), and the experimental process complied with relevant ethical standards.

### 2.2. Reagents

ABCA1 protein concentration determination kit (Batch Number 120720210702) was purchased from Biyuntian Biotechnology Co., Ltd. The CHO kit (Production Number 212072), TG kit (Production Number 218061), LDL kit (Production Number 221691), HDL kit (Production Number 221651), and glucose kit (Production Number [Glu] 213771) were purchased from Zhongsheng Beihang Biotechnology Co., Ltd. LCAT (Batch Number 20220902), CYP7A1 (Batch Number 20220902), HMGR (Batch Number 20220902), and PPAR*γ* (Batch Number 20220902) ELISA kits were purchased from Nanjing Jiancheng Biological Co., Ltd. Saturated Oil Red O dyeing solution (Lot Number MA0120) and the SDS–PAGE gel quick configuration box (Lot Number MA0159) were purchased from Dalian Meilun Biotechnology Co., Ltd. The PPAR*γ* antibody (Lot No. 0523R), ABCA1 antibody (Lot No. 60239 M), LXR*α* antibody (Lot No. 54023R), and sheep anti-mouse IgG enzymatically labelled secondary antibody (Lot No. 0295G) were purchased from Beijing Boosen Biotechnology Co., Ltd.

### 2.3. Main Instruments

The main instruments used in this study included the following: HYQ-2121 vortex mixer (Silent Shake), Tecan Infinite M200 Pro multifunctional micrometer (Tecan, Switzerland), 722 visible spectrophotometer (Shanghai Spectral Instrument Co., Ltd.), ISO 90C1 electronic balance (Beijing Sartorius Instrument System Co., Ltd.), 78HW-1 constant temperature magnetic stirrer (Jintan Medical Instrument Factory), Hitachi 7600-020 automatic biochemical analyzer (Hitachi High-Tech, Japan Co., Ltd.), HWS-150BX constant temperature and humidity box (Tianjin Test Instrument Co., Ltd.), and MIKRO 220R low-temperature centrifuge (German Hettich Company).

### 2.4. Drug Preparation

The “BYHWT” decoction consists of 120 g *Astragalus* root, 6 g Angelica tail, 3 g Sichuan Lovage Rhizome, 4.5 g Radix Paeoniae Rubra, 3 g peach kernel, 3 g *Carthamus tinctorius*, and 3 g earthworm. The Chinese medicinal slices used in the experiment were strictly purchased at the First Affiliated Hospital of Heilongjiang University of Chinese Medicine, in accordance with the species prescribed in the “Pharmacopoeia of the People's Republic of China 2020” and authenticated by Professor Xiao Hongbin of Heilongjiang University of Chinese Medicine.

The preparation of the “BYHWT” decoction is as follows: After mixing the medicinal herbs according to the prescription dosage, add 10 times the weight of distilled water, soak for 0.5 h, and then simmer for 1.5 h. Filter the obtained decoction and collect it. The residue is boiled again with eight times the weight of distilled water for 1 h, then filtered, and collected. Combine the two decoctions, and vacuum concentrate until the concentration is 1:1.4.

The extraction of different parts of the “BYHWT” decoction is as follows: Take a certain amount of the prepared decoction for separate preservation. To the remaining decoction, add ethanol and stir thoroughly with a glass rod until the final ethanol concentration in the decoction is 80%. Let it stand overnight, and then filter to separate the supernatant and precipitate. Dissolve the precipitate in a small amount of distilled water. Repeat the process three times, and dissolve the precipitate in a rotary evaporator after washing with different concentrations of ethanol. Concentrate the ethanol-deposited part under reduced pressure until the ethanol odor is not obvious. The obtained concentrated liquid is freeze-dried and stored as the “ethanol-deposited part.” Mix the supernatants obtained from the three times, concentrate under reduced pressure, and pass through a pretreated AB-8 macroporous resin column at a flow rate of 3 mL/min (0.12 BV/h) for 2 h. Use a gradient elution of distilled water, 30% ethanol, 50% ethanol, and 75% ethanol to collect the eluates. Remove ethanol under reduced pressure, freeze-dry the eluates, and name them “30% ethanol elution part,” “50% ethanol elution part,” and “75% ethanol elution part.” Store all parts at −20°C.

## 3. Methods

### 3.1. Animal Grouping, Modelling, and Treatment

After 1 week of adaptive feeding, all mice were randomly divided into 8 groups according to body weight: 10 mice were in the normal group, and the other 70 mice were randomly divided into the model group, fenofibrate group (fenofibrate), BYHWT group, alcohol precipitation group, 30% group, 50% group, and 75% group. In addition to the normal group, all other groups were fed a high-fat diet (diet (65%) + lard (10%) + sugar (15%) + milk powder (5%) + egg yolk powder (5%)). Intragastric administration of fenofibrate (26 mg/kg), BYHWT (14.0 g/kg), alcohol precipitation component (3.859 g/kg), and 30% (0.137 g/kg), 50% (0.122 g/kg), and 75% components (0.067 g/kg) began in the morning in a volume of 10 mL/kg. The normal group and model group were given an equal volume of distilled water once a day for 28 days (4 weeks).

### 3.2. Sampling Method

During the whole experimental period, the mental state, activity, hair colour, and food and water intake of the mice were observed every day. The food intake was measured daily, and the body mass was measured weekly. After 21 days of continuous administration, the mice were deprived of water for 12 h, and their fasting body mass was measured. Two hours after the last administration, mice in each group were anaesthetized by intraperitoneal injection of 0.4% pentobarbital sodium solution (50 mg/kg), and blood from the fundus venous plexus was taken, placed in an EP tube, left at room temperature for 2 h, and centrifuged at 3000 r/min for 10 min. Serum was separated, and blood lipid levels were detected by an automatic biochemical analyzer. After a sacrifice by cervical dislocation, the liver, spleen, spealbone fat, and epididymal fat were removed from the mice and weighed. The changes in liver/spleen/spealbone fat/epididymal fat index were calculated according to the following formula: liver/spleen/spealbone fat/epididymal fat index = liver/spleen/spealbone fat/epididymal fat mass (g)/body mass (g) × 100%. Subsequently, the same part of the liver was cut and frozen at −80°C. Part of the liver tissue homogenate was analyzed with an ELISA kit, part by qRT–PCR, and part by Western blot. Another part of the liver tissue was fixed with 10% neutral formalin and subjected to HE staining and Oil Red O staining. The epididymal fat was extracted and fixed with 10% neutral formalin for HE staining.

### 3.3. Methods of Drug Composition Analysis

Samples (20 ± 5 mg) from the alcohol precipitation group, 30% group, 50% group, and 75% group were accurately weighed into 2-mL centrifuge tubes. Then, 200 *μ*L of BYHWT stock was added to a 1.5-mL centrifuge tube, and 6-mm-diameter grinding beads and 400 *μ*L of each extract were added. The extraction solution was an 80% (*v*/*v*) methanol aqueous solution containing four internal standards (0.02 mg/mL 2-chloro-L-phenylalanine, etc.). A frozen tissue grinder was used for 6 min (−10°C, 50 Hz), low-temperature ultrasonic extraction was performed for 30 min (5°C, 40 KHz), and static incubation was performed for 30 min at −20°C, followed by centrifugation at 13,000 g for 15 min and 4°C. Finally, the supernatant was taken for analysis.

The chromatographic conditions were as follows. Mobile Phase A consisted of 95%water + 5%acetonitrile (containing 0.1% formic acid), and Mobile Phase B consisted of 47.5%acetonitrile + 47.5%isopropyl alcohol + 5%water (containing 0.1% formic acid). The column temperature was 40°C, the flow rate was 0.4 mL/min, and the injection volume was 3 *μ*L.

The mass spectrometry conditions were as follows. An electrospray ionization (ESI) source was used. Positive and negative ion scanning modes were used to collect the signals (m/z range 70–1050). Additionally, the sheath gas flow rate was 50 arb, the auxiliary gas flow rate was 13 arb, the source temperature was 425°C, and the capillary temperature was 325°C. The positive mode electrospray voltage was 3500 V, the negative mode electrospray voltage was −3500 V, the S-Lens voltage was 50, and the collision energy was set to 20, 40, and 60 eV. Finally, the resolution was 60,000.

## 4. Detection of Various Indices of the Mice

### 4.1. Detection of Serum Biochemical Indexes

Blood was taken from the fundus venous plexus of the mice, placed into an EP tube, left at room temperature for 2 h, and centrifuged at 3000 r/min for 10 min. The serum was separated and analyzed with an automatic biochemical instrument for TC, TG, LDL, and HDL contents following the instructions of the corresponding assay kit.

### 4.2. Detection of CHO Synthesis and Metabolism Indexes

The contents of LCAT, CYP7A1, and PPAR*γ* in liver homogenate were determined by ELISAs.

### 4.3. Haematoxylin–Eosin (HE) Staining Was Used to Detect the Pathological Morphology of the Liver Tissue and Fat

After the mice were sacrificed, the liver tissue and fat were taken, fully washed with precooled normal saline, fixed with 10% neutral formalin, dehydrated, cleared, waxed and embedded, and finally prepared into sections with a thickness of 5 *μ*m. For dewaxing, xylene, 50% xylene, anhydrous ethanol, and 95%, 90%, 85%, 80%, and 70% ethanol were used. After dewaxing, HE staining was performed, and the pathological changes in the liver and adipose tissue were observed under a microscope.

### 4.4. Oil Red O Staining

Fix the prepared frozen sections in 10% neutral formalin for 10 min and wash thoroughly with distilled water. (1) The slices were washed with 50% ethanol after drying. (2) Oil Red O ethanol staining solution was added for 8 min. (3) Differentiation was performed with 50% ethanol and terminated with tap water. (4) Haematoxylin was used to stain the nuclei, and the slices were washed with tap water until they appeared blue and sealed in glycerin gelatine.

### 4.5. Real-Time Fluorescence Quantitative PCR: Liver Tissue Was Taken to Determine the Expression of LCAT, PPAR*γ*, and CYP7A1

Appropriate amounts of liver tissue were taken, total RNA was extracted, the content and purity of the RNA were determined, and cDNA was synthesized by reverse transcription. The PCR amplification reaction conditions were as follows: 95°C, 10 min, one cycle and 95°C, 15 s, 60°C, 1 min, a total of 40 cycles. Using ACTB as the internal reference, the 2‐ΔΔCT method was used to quantitatively analyze the target genes. The primers were synthesized by Jilin Province Kumi Biotechnology Co., Ltd. and are listed in Table 1.

### 4.6. Western Blot: Liver Tissue Was Taken to Determine the Protein Expression Levels of PPAR*γ*, LXR*α*, and ABCA1

Frozen liver tissue (100 mg) was cut into pieces, and the tissue protein cleavage solution was added. After grinding with a grinder, the supernatant was centrifuged at 4°C and 13,000 r min^−1^ (centrifugation radius of 9.2 cm) for 10 min. The protein concentration was quantitatively determined by the BCA method. A protein loading buffer was added, and denaturation was performed at 95°C. The proteins in the gel were separated by SDS–PAGE and transferred to PVDF membranes. The samples were sealed with sealing liquid in a shaker at room temperature for 1 h. The primary antibody (1:1 000) was added, and the samples were incubated at 4°C overnight and washed with TBST three times. Each sample was then incubated with the specific secondary antibody (1:5000) at room temperature for 2 h, and the membrane was again washed with TBST three times. An imaging system was used for exposure, development, and photography. Photoshop was used to clean and remove the colour, and Alpha Software was used to calculate the optical density of each strip. Using *β*-actin as the internal reference, the target protein was semiquantitatively analyzed.

### 4.7. Data Processing

Excel 2019 was used to collect the data from each test index. SPSS Statistics 22.0 statistical analysis software and one-way ANOVA were used for statistical analysis.

## 5. Results

### 5.1. Drug Composition Analysis

#### 5.1.1. Analysis of the Composition of BYHWT

**Figure 1:**
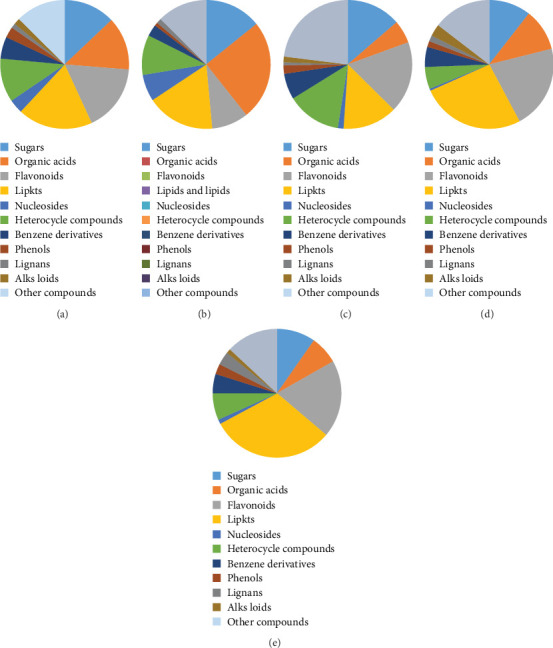
(a–e) The analysis of the composition of Buyang Huanwu Tang liquid, Buyang Huanwu Tang alcohol precipitation component, Buyang Huanwu Tang 30% component, Buyang Huanwu Tang 50% component, and Buyang Huanwu Tang 75% component. LMS analysis and database searches were performed, and the matching compounds were identified. (a) BYHWT; (b) alcohol precipitation; (c) 30%; (d) 50%; (e) 75%.

According to LC–MS and database searching and matching, a total of 257 compounds meeting the requirements were obtained, including 33 sugars, 34 organic acids, 43 flavonoids, 48 lipids and lipids, 9 nucleosides and nucleotides, 28 heterocyclic compounds, 14 benzene derivatives, 7 phenols, 3 lignans, 4 alkaloids, and 26 glycosides. There are also 32 other compounds. The proportions of various compounds are shown in Figure 1a.

#### 5.1.2. Analysis of the Components in the Alcohol Precipitation Component of BYHWT

According to LC–MS and database searching and matching, a total of 239 compounds were identified, including 34 carbohydrates, 60 organic acids, 22 flavonoids, 41 lipids and lipids, 16 nucleosides and nucleotides, 24 heterocyclic compounds, 7 benzene derivatives, 2 phenols, 3 alkaloids, and 30 other compounds. The proportions of various compounds are shown in Figure 1b.

#### 5.1.3. Analysis of the Components in the 30% Component of BYHWT

According to LC–MS and database searching and matching, a total of 159 compounds were identified, including 19 sugars, 28 organic acids, 25 flavonoids, 19 lipids and lipids, 2 nucleosides and nucleotides, 19 heterocyclic compounds, 9 benzene derivatives, 3 phenols, 1 lignan, and 2 alkaloids. There are also 32 other compounds. The proportions of various compounds are shown in Figure 1c.

#### 5.1.4. Analysis of the Components in the 50% Component of BYHWT

According to LC–MS and database searching and matching, a total of 199 compounds were identified, including 21 sugars, 21 organic acids, 42 flavonoids, 52 lipids and lipids, 1 nucleoside and nucleotide, 11 heterocyclic compounds, 10 benzene derivatives, 3 phenols, 3 lignans, and 6 alkaloids. There were 29 other compounds. The proportions of various compounds are shown in Figure 1d.

#### 5.1.5. Analysis of the Components in the 75% Component of BYHWT

According to LC–MS and database searching and matching, a total of 268 compounds were identified, including 26 sugars, 19 organic acids, 52 flavonoids, 83 lipids and lipids, 3 nucleosides and nucleotides, 18 heterocyclic compounds, 13 benzene derivatives, 7 phenols, 9 lignans, and 3 alkaloids. There were 35 other compounds. The proportions of various compounds are shown in Figure 1e.

We use methods such as water extraction, alcohol precipitation, and large-pore resin adsorption to separate the components of the Bu Yang Huan WuTang. Through LC–MS analysis and database searching and matching, we identify the main components in each component.

### 5.2. Lipid Content and Liver Function in a Hypercholesterolemia Mouse Model

**Figure 2:**
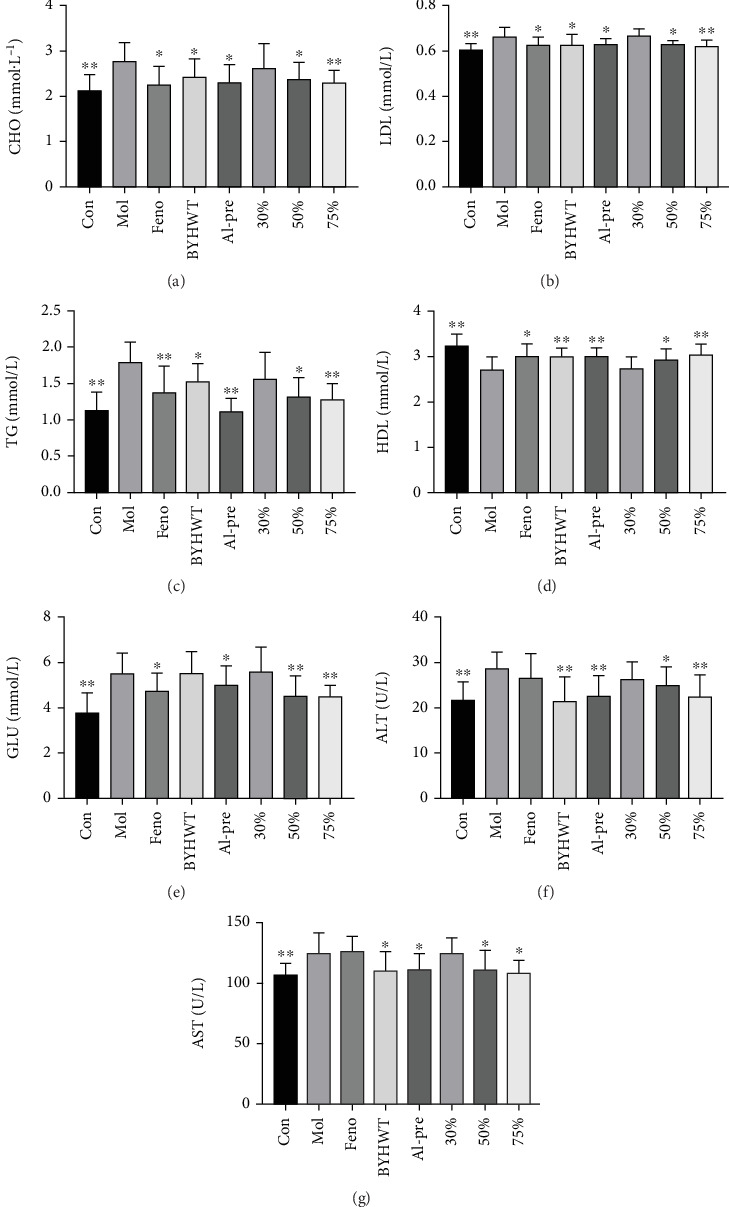
(a–g) The contents of CHO, LDL, TG, HDL, Glu, ALT, and AST in the serum of each group of mice (*n* = 10; ⁣^∗^*p* < 0.05, ⁣^∗∗^*p* < 0.01). After the mice were sacrificed, the blood samples were centrifuged, and serum TG, LDL, CHO, Glu, ALT, AST, and HDL were detected. (a) CHO; (b) LDL; (c) TG; (d) HDL; (e) Glu; (f) ALT; (g) AST.

Compared with the normal control group, the contents of TG, LDL, CHO, and Glu in the serum of the model control group were significantly increased (*p* < 0.05, *p* < 0.01), while the content of HDL was significantly decreased (*p* < 0.05, *p* < 0.01), indicating that hypercholesterolemia model was successfully constructed. Compared with the model control group, the CHO contents in the fenofibrate group, alcohol precipitation group, and 75% group were significantly decreased (*p* < 0.05, *p* < 0.01), the TG and LDL contents were significantly decreased (*p* < 0.05, *p* < 0.01), and the HDL content was significantly increased (*p* < 0.05, *p* < 0.01). The contents of HDL in the 30% group were not significantly increased (*p* > 0.05), and the contents of CHO, TG, LDL, Glu, ALT, and AST were not significantly decreased (*p* > 0.05). ALT and AST in the fenofibrate group showed an upward trend, as shown in Figures 2a, 2b, 2c, 2d, 2e, 2f, and 2g.

Blood was taken from the fundus venous plexus of the mice and placed into an EP tube. The serum was separated and analyzed with an automatic biochemical instrument for TC, TG, LDL, and HDL contents following the instructions of the corresponding assay kit.

### 5.3. Liver, Spleen, Spealbone, and Epididymis Fat Indices of the Hypercholesterolemia Mouse Model

**Figure 3:**
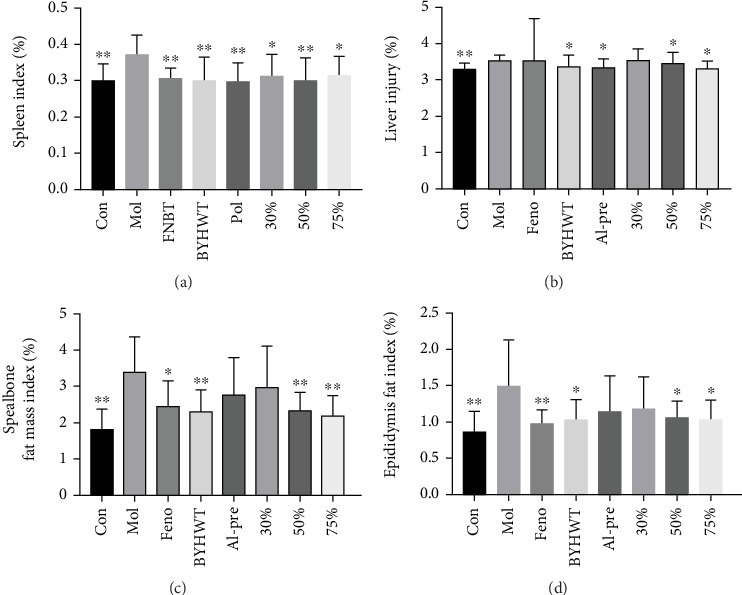
(a–d) The liver, spleen, spealbone fat, and epididymal fat indices from each group (*n* = 10; ⁣^∗^*p* < 0.05, ⁣^∗∗^*p* < 0.01). The fat from the liver, spleen, spealbone, and epididymis was weighed, and each index was calculated according to the formula above. (a) Spleen index; (b) liver injury; (c) spealbone fat mass index; (d) epididymis fat index.

The final body mass and weights of the liver, spleen, spealbone fat, and epididymal fat were determined before sampling, and the changes in the liver, spleen, spealbone fat, and epididymal fat indices were calculated according to the given formula. The results are shown in Figures 3a, 3b, 3c, and 3d. Compared with those in the model group, the weights of the liver, spleen, spealbone fat, and epididymal fat in each treatment group were significantly increased, and the indices were also significantly increased (*p* < 0.05, *p* < 0.01).

When obtaining the materials, we weigh the complete liver, spleen, spealbone fat, and epididymal fat. Through calculations using a specific formula, we calculate the organ indices of each organ for comparison.

### 5.4. ELISA Kits Were Used to Detect the Contents of LCAT, CYP7A1, and PPAR*γ* in Liver Homogenates

**Figure 4:**
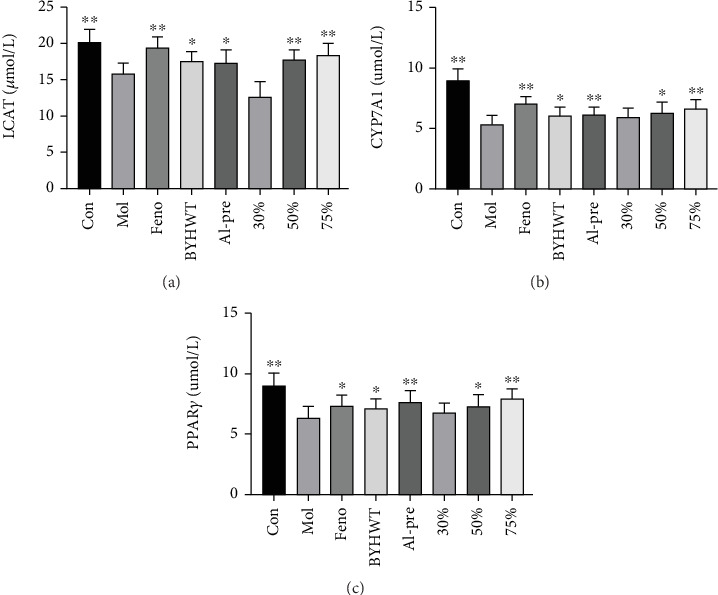
(a–c) The contents of LCAT, CYP7A1, and PPAR*γ* in the liver tissue of mice in each group (*n* = 10; ⁣^∗^*p* < 0.05, ⁣^∗∗^*p* < 0.01). The contents of LCAT, CYP7A1, and PPAR*γ* in liver homogenate were detected by ELISA kits. (a) LCAT; (b) CYP7A1; (c) PPAR*γ*.

The levels of LCAT, CYP7A1, and PPAR*γ* in the liver tissue of the mice in the model group were significantly lower than those in the normal group (*p* < 0.05, *p* < 0.01), and the above indices were significantly improved in all treatment groups, except the 30% group, compared with those in the model group (*p* < 0.05, *p* < 0.01), as shown in Figures 4a, 4b, and 4c.

We take 100 mg of liver tissue, grind it, centrifuge it to obtain the supernatant, and then use the reagent kit to detect the levels of LCAT, CYP7A1, and PPAR*γ* in the liver tissue according to the kit instructions.

### 5.5. HE Staining Was Used to Detect the Pathological Morphology of Liver and Fat Tissue

**Figure 5:**
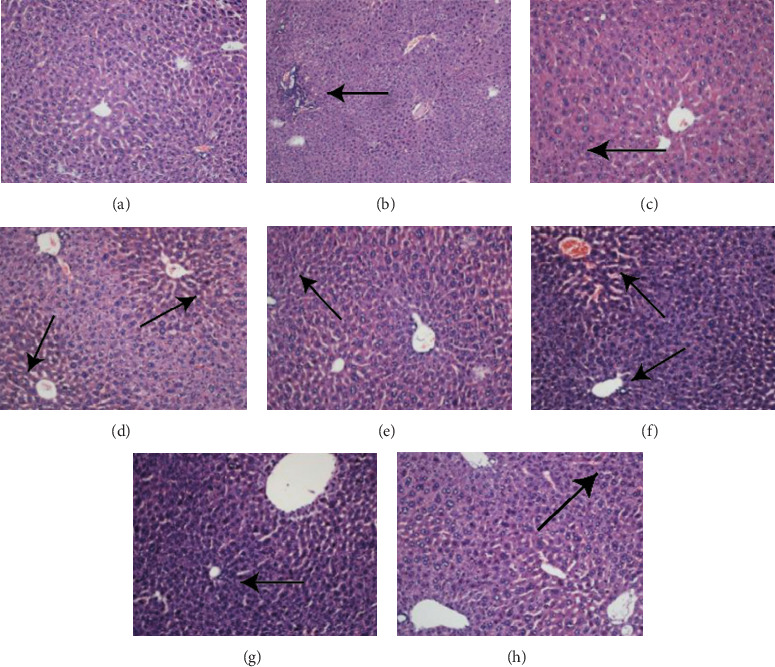
(a–h) The results of HE staining of the liver tissue from mice in each group. Liver tissue was taken and fixed with formalin, and then, HE staining was performed. (a) Normal group; (b) model group; (c) fenofibrate; (d) BYHWT; (e) alcohol precipitation; (f) 30%; (g) 50%; (h) 75%; images are ×200. The arrow indicates the location of the lesion.

The HE-staining images show that the liver cells in the normal control group were arranged in an orderly manner (Figure 5a) and were uniform in size with a full cytoplasm; moreover, no red lipid droplets were found in the hepatocytes, the nuclei were blue, and the structure of the hepatic sinuses was normal. In the model group, there were significant lipocyte translocation vacuoles (Figure 5b) and nuclear deformities, and cell volume and spacing showed a loose arrangement with a large number of infiltrating inflammatory cells. In the alcohol precipitation and 75% groups, there were fewer red lipid droplets in the liver cells, the structure of the hepatic sinusoids was restored, and the degree of lipid accumulation was reduced (Figures 5c, 5d, 5e, 5f, 5g, and 5h).

After weighing the liver, a portion of the liver is cut and fixed in 10% formalin. Following the steps of the reagent kit, we conduct an HE staining, observing the histopathological changes in the livers of different groups of mice.

**Figure 6:**
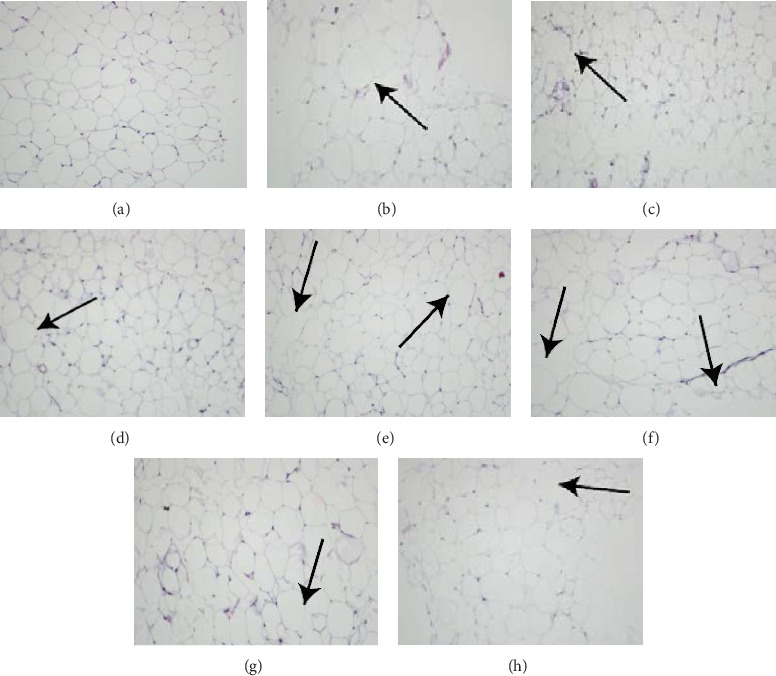
(a–h) The HE-staining images of adipose tissue from the epididymides of mice in each group. Adipose tissue was taken from the epididymis and fixed in formalin followed by staining with HE. (a) Normal group; (b) model group; (c) fenofibrate; (d) BYHWT; (e) alcohol precipitation; (f) 30%; (g) 50%; (h) 75%; images are ×200. The arrow indicates the location of the lesion.

The HE-staining images of adipose tissue (Figures 6a, 6b, 6c, 6d, 6e, 6f, 6g, and 6h) show that the adipose cells in the normal group were full, complete, and tightly structured. The adipocytes in the model group had a larger volume and more gaps and were broken. There was no significant change between the 30% group and the model group and no significant pathological changes between the alcohol precipitation and 75% groups.

After weighing the adipose tissue from the epididymides, a portion of the adipose tissue from the epididymides is cut and fixed in 10% formalin. Following the steps of the reagent kit, we conduct an HE staining, observing the histopathological changes in the livers of different groups of mice.

### 5.6. Lipid Deposition in the Liver of Mice in Each Group Was Observed by Oil Red O Staining

**Figure 7:**
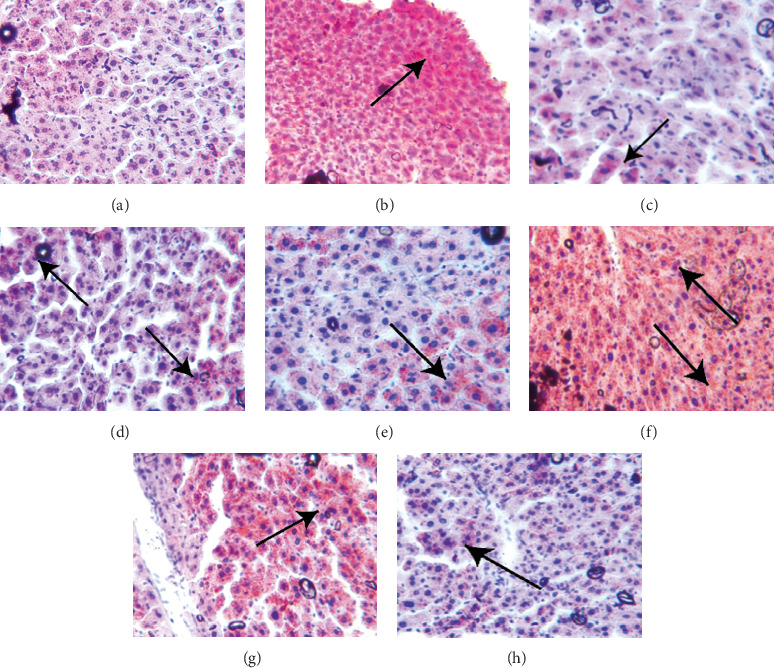
(a–h) The Oil Red O staining images to detect lipid deposition in the livers of mice in each group. Fresh liver tissue was taken and fixed in formalin, frozen sections were made into slices with a thickness of 10 *μ*m, and Oil Red O staining was performed. (a) Normal group; (b) model group; (c) fenofibrate; (d) BYHWT; (e) alcohol precipitation; (f) 30%; (g) 50%; (h) 75%; images are ×200. The arrow indicates the location of the lesion.

In the normal control group, a few orange–red lipid droplets (Figure 7a) were found in the hepatocytes, and the hepatocyte space was clear. In the model control group (Figure 7b), diffuse lipid droplets were observed in the hepatocytes of the mice, which were scattered throughout the orange–red visual field, with damaged nuclei and obvious deposition of lipids in the liver. Compared with the model group, there were fewer lipid droplets in the hepatocytes of all treatment groups (Figures 7c, 7d, 7e, 7f, 7g, and 7h) except for the 30% group.

Fresh liver tissue was fixed with 10% neutral formalin, and frozen sections were made into 10 *μ*m thick slices. Stain the liver according to the reagent kit instructions and observe the hepatic lipid droplets in the livers of various groups of mice.

### 5.7. Real-Time Fluorescence Quantitative PCR: Liver Tissue Was Taken to Determine the Expression of PPAR*γ*, LXR*α*, CYP7A1, and ABCA1

**Figure 8:**
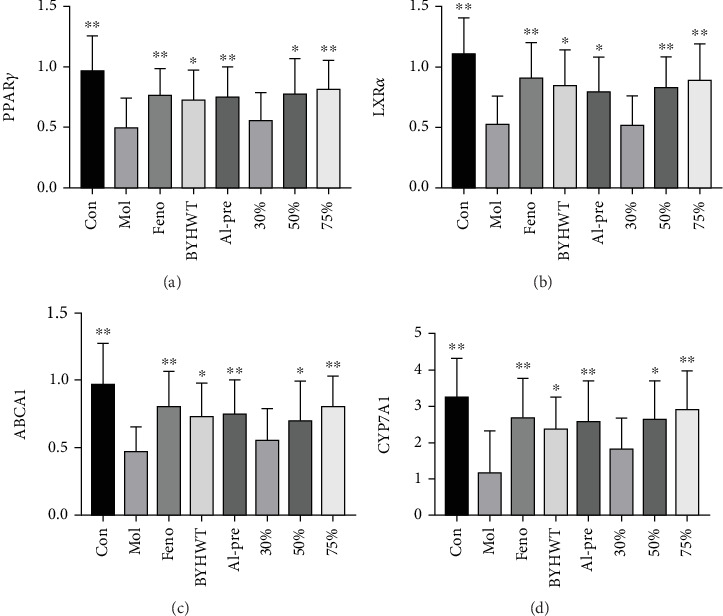
(a–d) The expression of PPAR*γ*, LXR*α*, CYP7A1, and ABCA1 in the liver tissue from mice in each group. Total RNA was extracted from liver tissue, the content and purity of the RNA were determined, and cDNA was synthesized by reverse transcription. Using ACTB as the internal reference, the 2‐ΔΔCT method was used to quantitatively analyze the target genes. (a) PPAR*γ*; (b) LXR*α*; (c) ABCA1; (d) CYP7A1.

The protein expression levels of PPAR*γ*, LXR*α*, CYP7A1, and ABCA1 in the liver tissue of the model group were significantly lower than those of the normal group (*p* < 0.05, *p* < 0.01). Protein expression in the alcohol precipitation and 75% groups was significantly lower than that in the model group (*p* < 0.05, *p* < 0.01); see Figures 8a, 8b, 8c, and 8d.

Appropriate amounts of liver tissue were taken, total RNA was extracted, the content and purity of the RNA were determined, and cDNA was synthesized by reverse transcription. Detect the levels of PPAR*γ*, LXR*α*, CYP7A1, and ABCA1 RNA in the livers of various mouse groups using a reagent kit.

### 5.8. Effects of BYHWT and Its Different Components on the Expression of PPAR*γ*/LXR*α*/ABCA1 Signalling Pathway-Related Proteins

**Figure 9:**
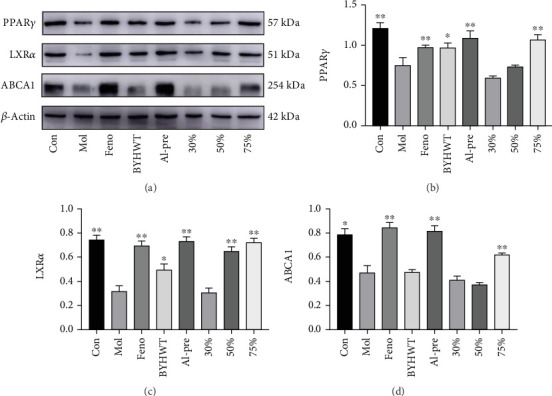
(a) The Western blot of PPAR*γ*/LXR*α*/ABCA1 signalling pathway-related protein expression in the liver tissue of mice in each group. (b–d) The protein expression levels of PPAR*γ*, LXR*α*, and ABCA1 in the liver tissue of mice in each group.

The expression of PPAR*γ*/LXR*α*/ABCA1 signalling pathway-related proteins is shown in the following figure. Compared with the model group, the protein contents of PPAR*γ*, LXR*α*, and ABCA1 in the liver tissue of the mice in the alcohol precipitation group and 75% group were significantly increased (*p* < 0.05), and the expression of LXR*α* in the 50% group was also significantly increased. The protein expression levels of PPAR*γ*, LXR*α*, and ABCA1 in the 30% group increased, but the difference was not significant (*p* > 0.05); see Figures 9a, 9b, 9c, and 9d.

Frozen liver tissue (100 mg) was cut into pieces, and the tissue protein cleavage solution was added. After grinding with a grinder, prepare tissue proteins using a reagent kit and analyze the levels of PPAR*γ*, LXR*α*, and ABCA1 proteins in the livers of various mouse groups using WB analysis.

## 6. Discussion

Hypercholesterolemia has a high incidence rate and can also cause many complications, including peripheral artery disease, which comes with a high rate of disability, stroke, and other high-risk diseases [15]. Traditional Chinese medicines are integrated and compatible with one another, giving these materials superior pharmacological effects in the treatment of hypercholesterolemia [16]. It has been reported that one-third of adults in developed countries and more than 80% of people in developing countries use herbs to treat diseases [17]. By regulating homeostasis in the body, Chinese herbs, such as *Astragalus* [18] and Angelica [19], have been confirmed to regulate CHO through different pathways and improve hypercholesterolemia. Ma et al. [20] used baicalein to regulate the PPAR*γ*/LXR*α*/ABCA1 signalling pathway to reduce liver function injury and inflammatory infiltration and improve glucose and lipid metabolism and insulin resistance in NAFLD rats. Isoflavones from tempeh can participate in the regulation of lipid metabolism and reduce inflammation by regulating the PPAR*γ*/LXR*α*/ABCA1 signalling pathway, thereby reducing lipid deposition in the liver. In our study, we found that the BYHWT whole prescription, alcohol precipitation component, and 50% and 75% components can ameliorate lipid disorders and regulate lipid levels to varying degrees, depending and are dependent on the activation of the PPAR*γ*/LXR*α*/ABCA1 signalling pathway.

In our study, an HFD significantly improved the blood lipid levels in hypercholesterolemia mouse model, which was reflected by significant increases in body weight, LDL, CHO, and TG levels and the decrease in HDL levels, among which the level of CHO [21] and the decrease in HDL were closely related to the feeding time and composition of the HFD. The HFD used in this experiment was composed of basic feed, lard, egg yolk powder, CHO, etc. The increase in serum HDL levels in mice was accompanied by a decrease in CHO levels, and the serum HDL level is an important indicator by which the promotion of CHO transfer from peripheral tissue to the liver for catabolism can be evaluated [22]. Therefore, in this experiment, we successfully established a mixed hypercholesterolemia model. After testing, the alcohol precipitation and 75% components had significant effects on improving blood lipid levels. At present, ALT and AST are generally regarded as the main indicators of liver function injury [23]. We detected ALT and AST levels and found that fenofibrate specifically reduced the CHO function; however, we found that the serum ALT and AST levels in the fenofibrate group increased. The alcohol precipitation and 75% ethanol treatments reduced ALT and AST levels and improved the severity of liver injury.

Studies have shown that reverse cholesterol transport (RCT) can effectively reduce intracellular CHO accumulation [24–26]. There are three proteins closely related to RCT: PPAR*γ*, ABCA1, and LXR*α*. PPAR*γ* is mainly present in adipocytes and liver and muscle tissue [27]. PPAR*γ* is also a multifunctional nuclear fatty acid receptor that regulates cellular carbohydrate and lipid homeostasis. Activated PPAR*γ* inhibits inflammation by inhibiting NF-*κ*B activity. LXR*α* can regulate the expression of lipid metabolism genes, thereby increasing CHO effluence and promoting CHO reversal [28]. ABCA1 is a member of the transporter family and is highly expressed in the liver, adrenal gland, brain tissue, and macrophage-derived foam cells. ABCA1 is both a lipid pump that can release CHO and phospholipids from cells and a transporter that mediates the uniaxial outflow of lipids from the cell membrane to extracellular receptors. PPAR*γ*/LXR*α*/ABCA1 signalling pathway activates and upregulates CYP7A1 expression, converting CHO esters in lipoproteins into bile acids and excreting them through feces [29].

A study found that nearly 30 factors affect the uptake, synthesis, metabolism, and excretion of CHO in the liver. CHO is absorbed from food absorption and is self-synthesized, and 70%–80% of this comes from the liver. In addition, RCT is the source and metabolic pathway of CHO and is the main factor affecting the level of CHO in the liver. The PPAR*γ*/LXR*α*/ABCA1 pathway is the main pathway involved in RCT [30]. PPAR*γ* is the upstream gene of LXR*α* [31]. When activated by PPAR*γ* in cells, LXR*α* can be promoted to bind to retinol-like X receptors, and this binding can increase the expression of ABCA1 [32, 33] and promote the reversal of CHO in peripheral tissues to the liver [34, 35]. ABCA1 plays a key role throughout the RCT process. ABCA1 transfers intracellular-free CHO and phospholipids to apolipoprotein A-I (ApoA-I), forming nascent disc-shaped pre-*β*-HDL, which continuously absorbs free CHO in peripheral tissues and achieves CHO transmembrane transport of CHO. Free CHO is converted into CHO esters by lecithin and CHO acyltransferase in HDL molecules, and these CHO esters form mature spherical HDL in HDL molecules [36–38]. CHO is transferred to lipoproteins by the action of cholesterol lipid transfer protein (CETP). PPAR*γ* and LXR*α* can activate and upregulate the expression of CYP7A1 to convert CHO lipids in lipoproteins into bile acids or excretion to be excreted in feces [39]. Therefore, these proteins are key factors regulating lipid metabolism. This study proved that the alcohol precipitation group and 75% group components can enhance RCT by upregulating the PPAR*γ*/LXR*α*/ABCA1 signalling pathway, thus reducing the lipid level in hypercholesterolemia mouse model and improving lipid accumulation in hepatocytes.

## 7. Conclusion

The underlying mechanism of CHO accumulation remains unclear. Therefore, the focus of this study was to investigate the mechanism of liver CHO accumulation induced by HFD in hypercholesterolemia mouse model and the effects of different components of BYHWT on the PPAR*γ*/LXR*α*/ABCA1 pathway in these mice. In our study, we found that the alcohol precipitation and 75% alcohol components significantly improved the pathological state of hypercholesterolemia in the model mice.

## Figures and Tables

**Table 1:** Primer sequence table.

**Primer**	**Sequence (5**⁣′**−3**⁣′**)**
ACTB-For	TCGTGCGTGACATCAAAGA
ACTB-Rev	AAGAAGGAAGGCTGGAAA
CYP7A1-For	TCATCACAAACTCCCTGTC
CYP7A1-Rev	TTTCCATCAXTTGGGTCTA
LCAT-For	GTGCTACCGTAAGACAGAGG
LCAT-Rev	CTTGCCAAAGCCAGGGACA
PPAR*γ*-For	TAGAACCTGCATCTCCACC
PPAR*γ*-Rev	CACAGACTCGGCACTCAAT

## Data Availability

All datasets generated for this study are included in the article material.
